# Mortality trends in primary malignant brain and central nervous system tumors vary by histopathology, age, race, and sex

**DOI:** 10.1007/s11060-023-04279-6

**Published:** 2023-03-16

**Authors:** Marisa Thierheimer, Gino Cioffi, Kristin A. Waite, Carol Kruchko, Quinn T. Ostrom, Jill S. Barnholtz-Sloan

**Affiliations:** 1 Case Western Reserve University, Cleveland, OH USA; 2 Division of Cancer Epidemiology and Genetics, Trans Divisional Research Program, National Cancer Institute, Bethesda, MD USA; 3 Central Brain Tumor Registry of the United States, Hinsdale, IL USA; 4 Department of Neurosurgery, Duke University School of Medicine, Durham, NC USA; 5 The Preston Robert Tisch Brain Tumor Center, Duke University School of Medicine, Durham, NC USA; 6 Duke Cancer Institute, Duke University Medical Center, Durham, NC USA; 7 Center for Biomedical Informatics and Information Technology, National Cancer Institute, Bethesda, MD USA

**Keywords:** Brain tumors, Central nervous system, Mortality trends, SEER

## Abstract

**Purpose:**

Primary malignant brain and other central nervous system tumors are rare cancers that have shown rising mortality rates in recent years. To elucidate potential factors involved in this rising death rate, we examined mortality trends for primary malignant BT in the United States stratified by histopathology groupings, age, race, and sex.

**Methods:**

Mortality rates for demographic factors within primary malignant BT were generated using the National Center for Health Statistics' National Vital Statistics Systems data from 2004 to 2018. Additionally, histopathology-specific incidence-based mortality rates were calculated using the National Cancer Institute’s Surveillance, Epidemiology, and End-Results (SEER) 18 data from 2004 to 2018. Joinpoint modeling was used to estimate mortality trends and annual percent changes with corresponding 95% confidence intervals.

**Results:**

Overall, there was a very small increase in mortality from 2004 to 2018. Individuals > 65 years saw a small increase in mortality, while changes in individuals of other ages were non-significant. Asian/Pacific Islander or American Indian/Alaskan Native had the largest increase in mortality. Among histopathology groupings, there was a small mortality increase in adults ages > 65 years with glioblastoma, while the mortality rate of other malignant gliomas declined in the same age group. CNS lymphoma mortality rates in patients ages 15–39 and 40–64 years declined significantly while rising significantly in the > 65 age group. In pediatric patients, embryonal tumor mortality had a non-significant increase between 2004 and 2007 but declined significantly between 2007 and 2018.

**Conclusion:**

Examining age, race, sex, and histopathology-specific mortality trends at the population level can provide important information for clinicians, researchers, and aid in public health planning.

**Supplementary Information:**

The online version contains supplementary material available at 10.1007/s11060-023-04279-6.

## Introduction

Population-based trends analysis provides insight into recent changes in mortality and can impact cancer prevention, surveillance and treatment. While overall cancer incidence rates (IR) and mortality rates have been decreasing, increases have been observed in some cancer types [1]. Due to their rarity, primary malignant brain and other central nervous system (CNS) tumors (collectively referred to here as BT) have often been excluded from large-scale mortality trend analyses despite being a significant source of morbidity and mortality. Primary malignant tumors comprised 29.1% of all newly diagnosed BT in 2014–2018. BT lead in overall cancer deaths among males < 40 years and females < 20 years [1].

Primary malignant BT had an average annual age-adjusted incidence rate (AAAIR) of 7.06 per 100,000 between 2014 and 2018 [2]. 5-year survival, while highly variable, was ~ 36%. From 1997 to 2017 the 5-year relative survival trend for BT improved ~ 1% while the overall cancer 5-year relative survival improved ~ 5%. It was estimated that in 2022 there would be 25,050 newly diagnosed, and 18,280 deaths from primary malignant BT [1].

Between 2013 and 2017, the incidence of BT decreased while death rates increased [3], prompting us to analyze mortality trends. Here we provide an updated analysis on overall mortality rate trends. Further, we perform additional analysis stratifying by age, race, sex, and histopathology in order to determine which histopathologies drive the observed changes over time in overall BT mortality and examine how trends for the major histopathologies differ among children and adults. Population based statistics, such as those described here, provide vital information on disease burden and characteristics to healthcare professionals, researchers, and patients.

## Methods

*Data Collection:* Mortality data from 2004 to 2018 were obtained from the National Center for Health Statistics’ (NCHS) National Vital Statistics System (NVSS), and the National Cancer Institute’s (NCI) Surveillance, Epidemiology, and End Results (SEER) program [4, 5]. NVSS data is derived from death certificates and aggregates mortality data for 50 States and the District of Columbia (100% of the US population). NVSS data contains limited individual-level data. Cases were classified by a cause of death (COD) from a malignant BT. The NVSS database was utilized to generate comprehensive mortality trend estimates in the US.

SEER18 data provides information from 18 population-based cancer registries, representing 28% of the US population [5]. SEER utilizes incidence-based mortality data, allowing trend estimates to be evaluated by specific histopathology and behavior codes for diagnosed cases. Cases for overall mortality rate analyses were classified by *the International Classification of Diseases for Oncology, Third Edition* (ICD-O-3) primary site codes [6]. For children (0–14 years), these groupings are defined using Supplementary Table 2 of the 2022 *Pediatric Brain Tumor Foundation CBTRUS Statistical Report* [7]. For all other ages, the histopathology groupings are defined in Table 1 of the 2022 CBTRUS Statistical Report [2].

*Statistical Analysis:* Annual mortality rates and incidence-based mortality rates were generated using SEER*Stat 8.4.0.1 [8]. All rates are presented per 100,000 population and were age-adjusted to the 2000 US standard population. Joinpoint 4.9.1.0 [9] was used to assess mortality rate trends using default settings: Two maximum Joinpoints, two minimum observations between two Joinpoints and end of data, 4499 number of permutations, and confidence intervals were calculated using the parametric method. Final Joinpoint models were selected using permutation tests. Annual percent changes (APC) are presented, along with the respective 95% confidence intervals (95% CI). Overall Age-adjusted mortality rate trends were generated in both NVSS and SEER18, as well as by age groups (0–14, 15–39, 40–64, 65 + years), sex (male, female), and race (White, Black, Other). Due to limited sample size, the “Other” category consists of Asian & Pacific Islander and American Indian & Alaska Native individuals.

To assess the potential influence of changes in incidence over time, age-adjusted mortality-to-incidence ratios (MIR) were calculated by age group using both the SEER18 incidence [10] and incidence-based mortality data from 2004 to 2018, utilizing the methodology described by Fay, using the ‘dsr’ function in package ‘heaven’ of R software (version 4.1.0) [5, 11, 12]. Joinpoint software was used to evaluate MIR trends, and APCs and associated 95% CI are reported. For Joinpoint analysis of MIR trends, MIR values were assumed to have constant variance.

All figures were generated using R Software (version 4.1.0). P values < 0.05 were considered statistically significant.

All analyses used cases diagnosed with a primary malignant brain tumor between 2004 and 2018 unless otherwise specified.

## Results

**Fig. 1:**
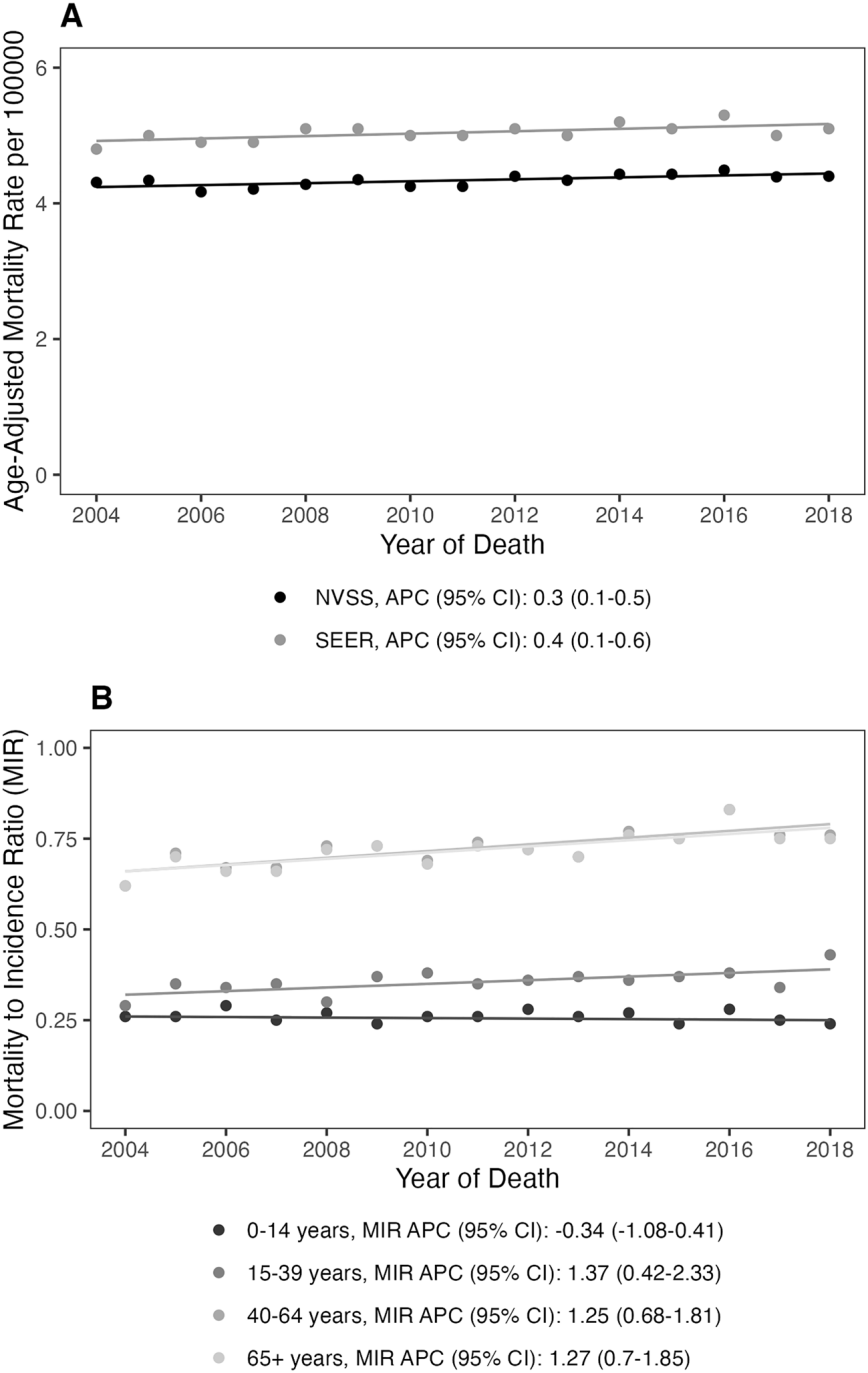
**A** Overall age-adjusted mortality rates and annual percent changes in primary malignant brain tumors from 2004 and 2018 in NVSS and SEER. **B** Mortality to incidence ratios and annual percent changes (APCs with 95% CI) in primary malignant brain tumors among children (0–14 years), adolescent young adults (15–39 years), adults (40–64 years), and older adults (65 + years) from 2004 and 2018 in SEER (APC: annual percent change; 95% CI 95% confidence interval)

**Fig. 2:**
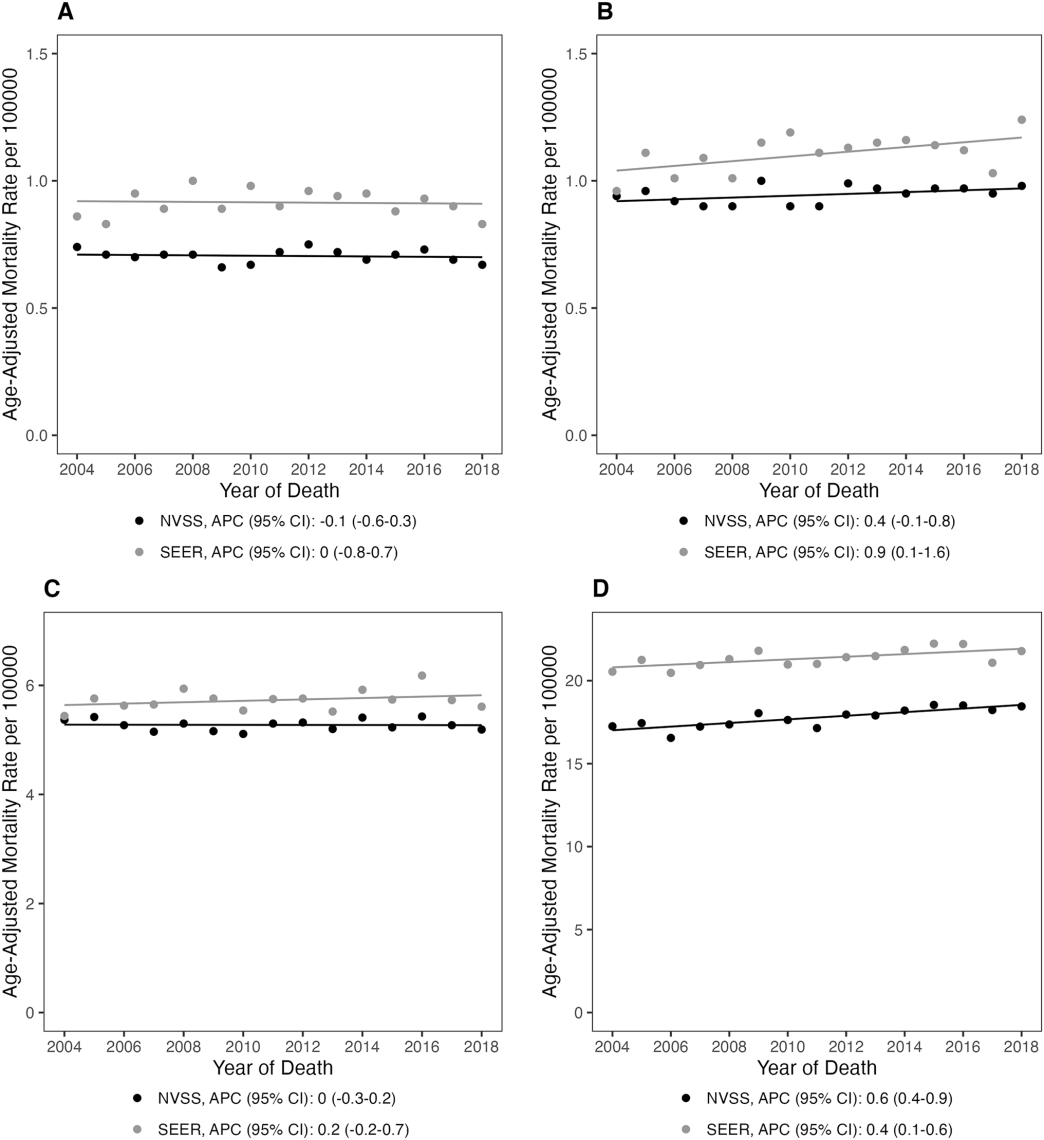
Age-adjusted mortality rates and annual percent changes in primary malignant brain tumors among **A** children (0–14 years), **B** adolescent young adults (15–39 years), **C** adults (40–64 years), and **D** older adults (65 + years) from 2004 and 2018 in NVSS and SEER (APC: annual percent change; 95% CI 95% confidence interval)

**Fig. 3:**
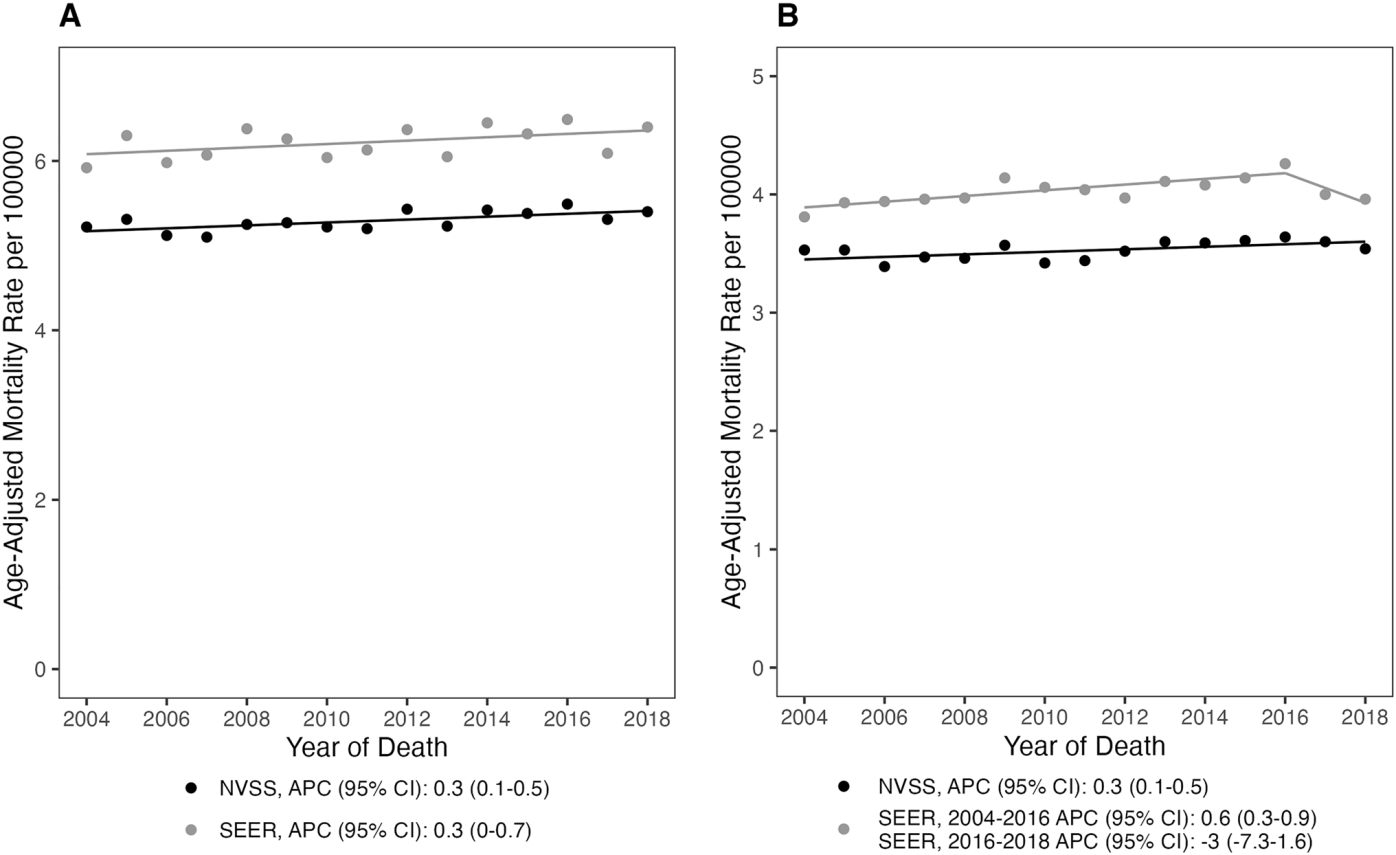
Age-adjusted mortality rates and annual percent changes in primary malignant brain tumors among **A** males, and **B** females from 2004 and 2018 in NVSS and SEER (APC: annual percent change; 95% CI 95% confidence interval)

**Fig. 4:**
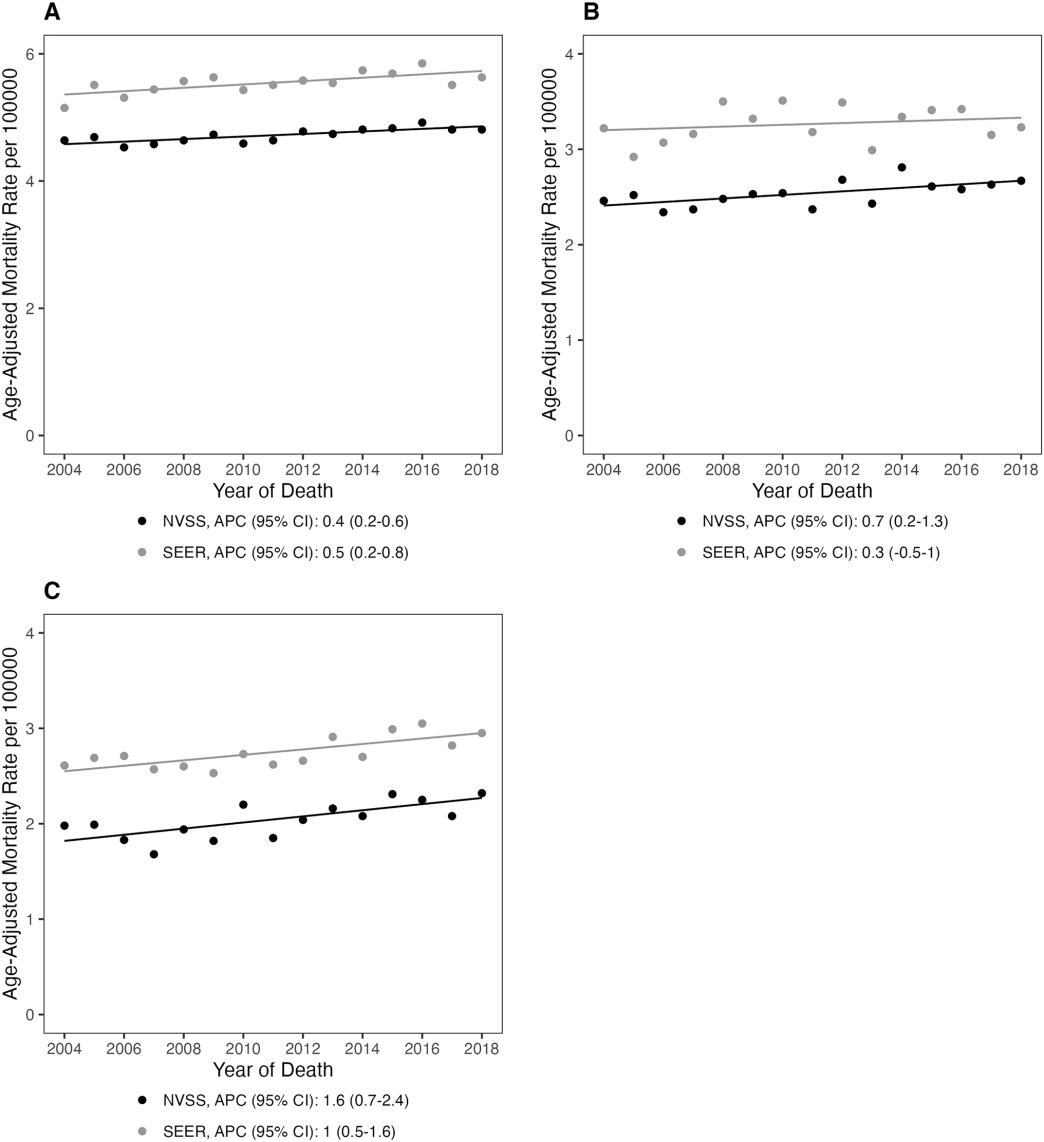
Age-adjusted mortality rates and annual percent changes in primary malignant brain tumors among **A** individuals who are White, **B** individuals who are Black, and **C** individuals of “other” race (Asian or Pacific Islander, and American Indian or Alaskan Native from 2004 and 2018 in NVSS and SEER (APC: annual percent change; 95% CI 95% confidence interval)

There was a small, yet significant, increase in overall BT mortality rate for both the NVSS and SEER18 data sets (APC = 0.3%, 95% CI 0.1% − 0.5%; APC = 0.4%, 95% CI 0.1% − 0.6%, respectively) (Fig. 1, Supplemental Table 1). Mortality rates from the NVSS and SEER18 datasets were consistently similar overall as well as across age (Fig. 2), sex (Fig. 3), and race (Fig. 4).

MIR were calculated in order to estimate the change in mortality due to primary malignant BT in relation to age-specific incidence. MIR was lowest in individuals aged 0–14 years, and highest in those individuals aged 65 + years. There was a small but significant increase in MIR over time in individuals aged 15–39, 60–64, and 65 + years (APC = 1.4%, 95% CI 0.4, 2.3; APC = 1.3%, 95% CI 0.7, 1.8; APC = 1.3%, 95% CI 0.7, 1.9, respectively) (Fig. 1, Supplemental Table 2).

There was significant variation in mortality rate trends by age, with mortality rates consistently increasing with age. Within the NVSS dataset, no significant increase in mortality rate was observed in individuals who died of a primary malignant BT between the ages of 15–39 years (APC = 0.4%, 95% CI − 0.1, 0.8). Individuals aged 40–64 years had negligible increases in mortality (APC = 0.0%, 95% CI − 0.3, 0.2) and those aged 65 + had a significant increase in mortality (APC = 0.6%, 95% CI 0.4, 0.9). Similar results were observed with the SEER18 dataset for individuals who were 15–39 years old (APC = 0.9%, 95% CI 0.1, 1.6) and those aged 40–64 years (APC = 0.2%, 95% CI − 0.2, 0.7). For those 65 years and older, the SEER18 dataset demonstrated a significant increase in mortality (APC = 0.4%, 95% CI 0.1, 0.6). In both datasets, individuals 0–14 years who died of a primary malignant BT experienced stable mortality rate trends (NVSS APC =  − 0.1%, 95% CI − 0.6, 0.3; SEER18 APC = 0.0%, 95% CI − 0.8, 0.7) (Fig. 2, Supplemental Table 1).

BT mortality rate was consistently higher among males than females. In both datasets there was a small, but statistically significant, rise in mortality in males (NVSS APC = 0.3%, 95% CI 0.1, 0.5; SEER18 APC = 0.3%, 95% CI 0.0, 0.7). In females, there was a small, yet statistically significant increase in mortality in the NVSS dataset (NVSS APC = 0.3%, 95% CI 0.1, 0.5). Within the SEER18 dataset, females had a significant increase in mortality from 2004 to 2016 followed by a statistically insignificant decrease from 2016 to 2018 (APC = 0.6%, 95% CI 0.3, 0.9; APC =  − 3.0%, 95% CI − 7.3, 1.6, respectively) (Fig. 3, Supplemental 1).

When stratifying by race, the mortality rate was highest in the “Other” racial group. Individuals who were White had a higher mortality rate compared to individuals who were. In the NVSS dataset, all three racial groups had statistically significant rises in mortality rate trends (“Other”: APC = 1.6%, 95% CI 0.7, 2.4; White: APC = 0.4%, 95% CI 0.2, 0.6; Black: APC = 0.7%, 95% CI 0.2, 1.3) (Fig. 3, Supplemental Table 1). In the SEER18 dataset, individuals who were Black had a statistically insignificant increase in mortality (APC = 0.3%, 95% CI − 0.5, 1.0) while individuals who were White experienced a small but statistically significant increase in mortality over time (APC = 0.5%, 95% CI 0.2, 0.8) and individuals in the “Other” race category had a significant increase (APC = 1.0%, 95% CI 0.5, 1.6) (Fig. 4, Supplemental Table 1).

**Fig. 5:**
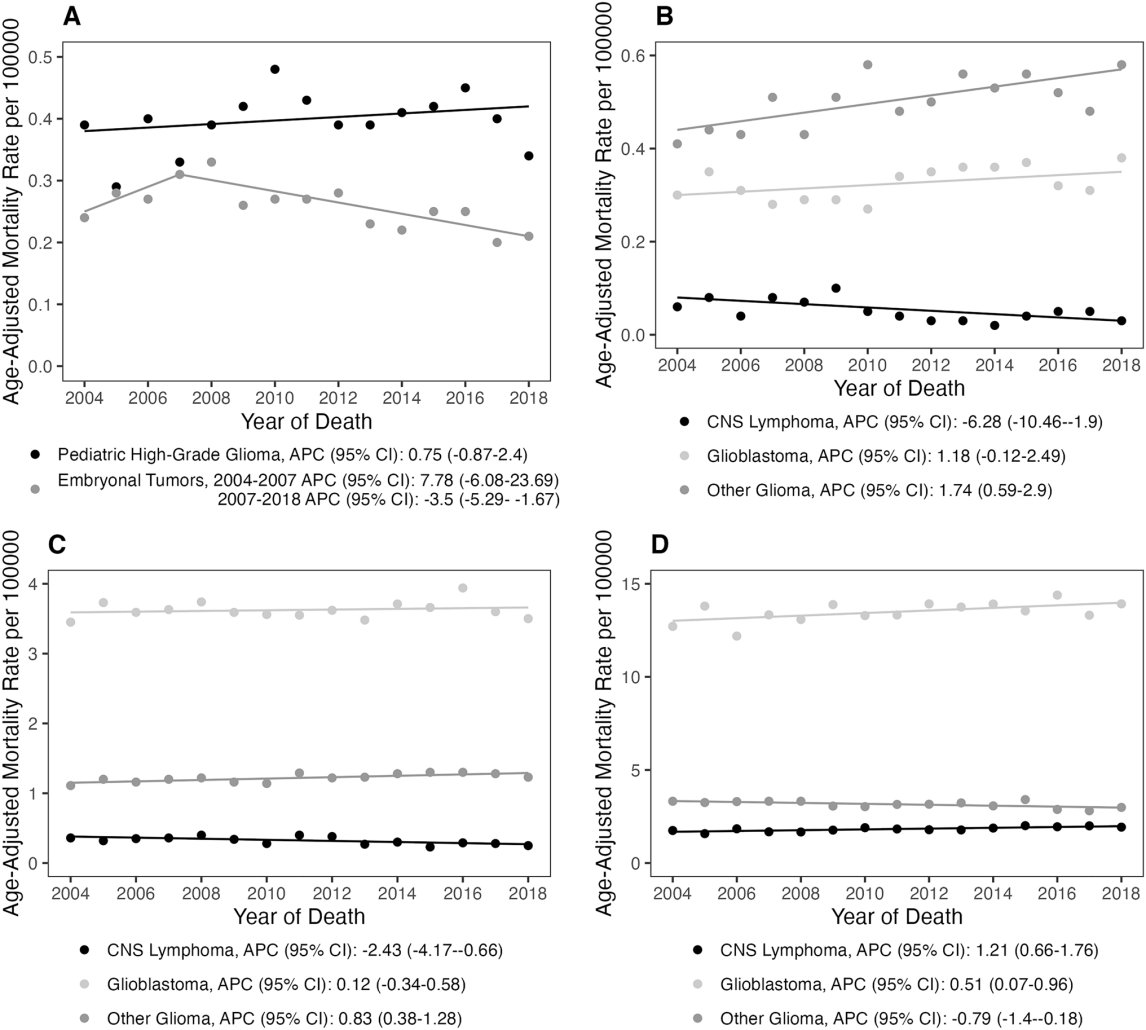
Histopathology specific age-adjusted mortality rates and annual percent changes among **A** children (0–14 years), **B** adolescent young adults (15–39 years), **C** adults (40–64 years), and **D** older adults (65 + years) from 2004 and 2018 (SEER) for the most common histopathologies by age group (APC: annual percent change; 95% CI 95% confidence interval)

Due to their large share of mortality in individuals 0–14 years, embryonal tumors and pediatric high-grade gliomas were examined in more detail. In the SEER18 dataset, children (0–14 years old) diagnosed with embryonal tumors experienced a large, yet statistically insignificant increase of 7.8% (95% CI − 6.1, 23.7) in mortality from 2004 and 2007. This was followed by a statistically significant decrease of − 3.5% (95% CI − 5.3, − 1.7) in mortality rates in the same population between 2007 and 2018 (Fig. 5, Supplemental Table 3). When examining mortality trends in individuals diagnosed with pediatric high-grade gliomas, there was a statistically insignificant increase between 2004 and 2018 (APC = 0.8%, 95% CI − 0.9, 2.4). Over the 15 years studied, the average APC showed a statistically insignificant mortality rate trend increase of 1.5% (95% CI − 3.1, 6.3) (Fig. 5, Supplemental Table 3).

For individuals aged 15 years and older, the following three histopathologies were analyzed in various age groups as well as in aggregate: glioblastoma, other malignant gliomas, and CNS lymphomas. These histopathologies were selected due to their large mortality case counts. Individuals 15 + years diagnosed with glioblastoma had a small, but significant, increase in mortality trends (APC = 0.4%, 95% CI 0.0, 0.8; SEER18 dataset). When examining glioblastoma mortality rates by age group, we found that individuals aged 15–39 years had an insignificant increase of 1.2% (95% CI − 0.1, 2.5), and individuals ages 40–64 years were relatively stable at 0.1% (95% CI − 0.3, 0.6), while individuals ages 65 + years had a small but significant increase (APC = 0.5%; 95% CI 0.1, 1.0). Examination of the mortality trends in individuals aged 15 + diagnosed with other malignant gliomas showed an insignificant increase in mortality trends (APC = 0.3%; 95% CI − 0.1, 0.7). When analyzing glioma mortality by age groups, there was a significant increase in mortality rate in individuals aged 15–39 years (APC = 1.7%; 95% CI 0.6, 2.9), a slight but statistically significant increase in individuals aged 40–64 years (APC = 0.8%, 95% CI 0.4, 1.3), and a small but significant decrease in those individuals aged 65 years and older (APC = − 0.8%; 95% CI − 1.4, − 0.2). For individuals aged 15 + years diagnosed with CNS lymphoma, the SEER18 dataset showed a small and variable decrease in mortality over time of -0.2% (95% CI − .8, 0.3). There was a significant decrease in CNS lymphoma mortality over time of − 6.3% (95% CI − 10.5, − 1.9) in individuals aged 15–39 years. Those individuals aged 40–64 years had a smaller, but nonetheless significant, decrease in mortality rate trends (APC = -2.4%; 95% CI − 4.2, − 0.7). In contrast, there was a significant increase in individuals aged 65 + years (APC = 1.2%; 95% CI 0.7, 1.8). (Fig. 5, Supplemental Table 3).

## Discussion

Improvements in life expectancy has propelled an increase in the older population. Further, In the US, birth rates are declining while those in the Baby Boom and Generation X are aging, resulting in increasing median age, which will continue to rise in the coming years. This study describes mortality trends among individuals with primary malignant BT in the context of aging demographics in the US population. While the mortality rates reported here reflect actual deaths due to disease, they cannot be separated from demographic patterns of the base population. The incidence rate of malignant primary brain and central nervous system tumors is highest in the elderly, at a rate more than three times higher than the general population [13]. The overall number of primary malignant brain tumor diagnoses and deaths will thus inherently rise with increases in the elderly segment of the population, which is growing faster than any other age group [14]. In support of this point, one study found that the highest numbers of primary malignant brain tumor diagnoses for the years 2021 and 2022 were predicted to be in individuals aged 65 years and older [2]. Due to the predicted increase in the elderly population combined with the known disease burden within this population, and those younger, it is important to maintain consistent reporting on mortality rates and trends.

Previous analysis of US primary malignant BT incidence trends from 2000 to 2010 found no substantial change in adults (≥ 20 years) and a small, but significant increase, in those < 20 years [15]. The NCI’s *Report to the Nation* reported that between 2013 and 2017 the incidence of malignant BT decreased while death rates increased [3]. Here, using two population-based databases, we found there was a small, but significant, increase in overall mortality from 2004–2018, which is consistent with previous reports (NVSS APC = 0.3%, 95% CI 0.1% − 0.5%,SEER18 APC = 0.4%, 95% CI 0.1% − 0.6%). It is notable that mortality rates are higher overall and among demographic groups in the SEER database than in NVSS. NVSS and SEER use different methodologies to collect mortality data, as well as different definitions for classifying primary BT, which may contribute to the discrepancy in the magnitude of mortality rate between databases. Despite these slight differences, these findings here demonstrate that the mortality rate trends from 2004–2018 are consistent between SEER and NVSS. Glioblastoma had the highest histopathology-specific increase in mortality rate for adults (APC = 0.4%, 95% CI 0.0, 0.8), while high-grade gliomas showed the largest increase in children aged 0–14 years (APC = 0.8%, 95% CI − 0.9, 2.4).

MIR was used to examine the direct relationship between mortality and incidence in order to account for potential screening bias. In principle, if changes in mortality are proportional to changes in incidence, MIR results should be consistent over time. However, an increase of MIR was observed across the study period for the SEER dataset, suggesting that at least a portion of observed large trends in mortality rates are not attributable solely to increased incidence. Rather, these are true increases in death rates in the specific demographic groups described. It is important to acknowledge the significant effect that incidence changes can have on mortality rates and the findings described here support the conclusions of previous research [16, 17].

While males and females showed similar increases in mortality rate over time, males consistently experienced higher mortality rates for each histopathology. Previous studies demonstrated that the incidence of primary malignant BT is significantly higher in males [2] likely leading to the increased mortality rates found here. Incidence rates for BT were highest in individuals who were White (7.55 per 100,000) compared to individuals who were either Black (4.44 per 100,000), AIAN (3.54 per 100,000), or API (4.40 per 100,000) [2], coinciding with overall mortality rate. Since 1975, the incidence of all cancers in the 0–14 and 15–39 year age groups have been increasing [18]. Concurrently, the mortality rate trends for all cancers in these age groups have declined by more than half between 1970 and 2019. Despite decreasing mortality rate trends, cancer remains the second-most common COD among children aged 1–14 years in the US, surpassed only by accidents, and is the fourth most common COD among individuals age 15–19 years [1].

BT are the second most common type of childhood cancer, and the most common cancer in those aged 15–19 years. In contrast to declining incidence trends in adults, the incidence rate of those 0–19 years old increased slightly between 2008 and 2017 [19]. This study, in agreement with the NCI’s *Report to the Nation*, only found a slight increase in mortality rate over time in this age group with the 0–14 age group remaining stable [3] despite increased incidence. While overall mortality due to BT has decreased over the last several decades, it still constitutes a greater proportion of overall cancer mortality in children as mortality in other cancer types has either slowly declined or remained stable. Childhood mortality due to acute lymphocytic leukemia, non-Hodgkin lymphoma, and Hodgkin lymphoma declined at a faster rate than malignant BT from 1975 to 2010 [15]. It would be expected that notable therapeutic advances has likely decreased mortality from other childhood cancers at a larger rate [1].

Though previous analyses showed overall mortality rates due to malignant BT in children and adolescents remained stable [17, 20], this study, when analyzing by histopathology, found statistically significant increasing mortality rate trends. Of note, there was a significant increase in mortality rate due to high grade glioma. In children, the most common BT histopathology was glioma (52.9%) and high-grade gliomas caused the greatest proportion of BT deaths (43.8%) [21]. Although advances in treatment have led to stabilizing and decreasing mortality rate trends, poor treatment outcomes continue to cause substantial morbidity and decreased quality of life for patients, particularly children and adolescents [22–25].

Contrary to a similar study [26], we found that mortality rate trends in glioma patients ages 40 + years remained stable. Our results corroborate a different analysis that showed incidence to be relatively stable in this patient population [20]. In the SEER18 dataset, there was a small but statistically significant increase in mortality rates over time due to glioblastoma among the elderly. These findings suggest that glioblastoma is a strong driver of overall BT mortality in this age group, as there was not a significant increase in mortality rate over time among other glioma histopathologies or non-glial brain tumors. However, this result may be confounded by the recent removal of isocitrate dehydrogenase (I*DH*) gene-mutant gliomas from the glioblastoma classification. Individuals with *IDH* gene-mutant gliomas have a better survival prognosis [27].

Although this study reports an increasing mortality rate among individuals with glioblastoma, continuing advances in treatment such as tumor-treating fields (TTF) have demonstrated promise in combination with existing therapies [28]. As these new treatments are only now becoming available, their impact on survival may not yet be readily observable. It will be imperative to continue to follow mortality trends in order to accurately assess the impact that these treatments will have on BT mortality as they become more widely accessible and utilized.

Older persons, especially those with comorbidities, may not be diagnosed and/or treated in the same manner as younger persons [29, 30]. A lack of diagnosis may lead to a delay or loss of recorded cases in prior years, potentially resulting in increased mortality due to delayed diagnosis. Mortality rates have increased during a time of significant advancement in diagnostic technology and treatment [31], yet aggressive treatments and clinical trials are less often recommended to elderly individuals. Additionally, older individuals have an increased risk of complications and treatment toxicity due to comorbidities, potentially leading to increased mortality [31]. Ongoing research, however, has shown that while being a strong predictor of survival, age is not the sole predictor for survival in certain BT, such as glioblastoma [32]. For patients with glioblastoma, performance status has been shown to have a greater impact on survival outcomes [33, 34].

While it is well-documented that males have higher incidence and mortality rates for BT, the underlying mechanism has not been elucidated [35, 36]. There is no apparent difference in time to treatment between males and females, however males have a higher proportion of glioblastoma and lower grade glioma and a higher risk of death compared to females [37]. Continuing to rigorously document the differences in incidence, mortality, and other clinical outcomes has the potential to improve upon sex-based approaches to brain tumor screening, assessment, and treatment.

There has been a statistically significant increase in the incidence of malignant BT between the mid-1970s and mid-1980s. These increases may be caused in part by screening bias due to the use of new medical imaging technologies [38]. In this current study, there was no increase in BT incidence. This may be because the most aggressive malignant tumors of the CNS, glioblastomas, have a slow dissemination process allowing structures to gradually adapt to both compression and deformation caused by the tumor mass. For this reason, even in the case of pronounced morphological signs of tumor penetration into brain tissue, clinical manifestations may be completely absent [39].

This study is not without limitations. The NVSS database relies on cause of death as recorded on the death certificate and does not specify the BT type. There is variation among states regarding procedures for deciding primary COD. Additionally, diagnostic technologies and histopathology definitions have evolved during 15-year period included in this study, potentially resulting in inconsistently recorded causes of death for similar cases [27, 40, 41]. The SEER18 dataset is limited by its reliance on incident cases to calculate mortality rate, and as a result, is highly sensitive to fluctuations in IR. This study is also affected by observation time bias which led to decreases in mortality rate at the beginning of the observation period due to the exclusion of cases diagnosed before the year 2000 in the SEER18 database. We attempted to correct for this bias by shifting the beginning of the study period to 2004, seeing as BT life expectancy after diagnosis was less than four years. Currently, there is no publicly available data source for the collection of survival and outcomes data from all geographic regions in the US via the cancer registry system. The SEER18 registries encompass less than one third of the US population and may represent areas of the US with increased access to medical resources potentially inflating screening bias. With the increased recognition of the value of biomarkers for specific BT histopathologies in classification, the WHO *Classification of Tumours of the Central Nervous System* has included biomarkers in its 2016 revision, potentially impacting the histopathological classification of tumors included in this analysis in 2017 and 2018 [27]. There is no mechanism for central pathology review within the SEER18 system, and histopathology code assignment at case registration is based on histopathology information contained in the patient’s medical record.

## Conclusion

Malignant BT cause significant mortality, disproportionate to their incidence. This study analyzed the most up-to-date data for malignant BT in the US, from 2004 to 2018, by age, race, histopathology, and sex. Overall, there was a very small increase in mortality over time, though some important significant changes were observed within specific age and histopathology groups. Mortality trends provide critical information regarding which groups carry the highest burden of primary BT in the US, as well as providing information that can be used to quantify the impact of advances in diagnosis and treatment. Therefore, trends should be periodically assessed in order to adequately assess advances as well as aid in public health planning. Future studies of BT mortality statistics should focus on up-and-coming advances in treatments as they evolve and become the standard of care. The potential areas of interest may include tumor treating fields, immunotherapy, and biomarker-based precision medicine.

## Supplementary Information

Below is the link to the electronic supplementary material.

Supplementary file1 (DOCX 18 KB)

## Data Availability

NVSS mortality and SEER incidence and incidence based mortality data is publically accessible and can be requested at the following website: https://seer.cancer.gov/data/access.html.
