# Supplementary material for: Mortality trends in primary malignant brain and central nervous system tumors vary by histopathology, age, race, and sex
Source: J Neurooncol. 2023 Mar 16;162(1):167–77. doi: 10.1007/s11060-023-04279-6 (PMC10050015; doi:10.1007/s11060-023-04279-6)
Supplement: Supplementary file 1 — Supplementary file1 (DOCX 18 KB) [file 11060_2023_4279_MOESM1_ESM.docx]

**Supplemental Table 1: Mortality trends in primary malignant brain tumors across demographic factors from 2004-2018 (NVSS and SEER) (APC: Annual Percent Change; 95% CI: 95% Confidence Interval)**

|  | **NVSS** | | | | **SEER** | | | | | | |
| --- | --- | --- | --- | --- | --- | --- | --- | --- | --- | --- | --- |
|  | **Trend 1** | | | | **Trend 1** | | | | **Trend 2** | | |
|  | **Years** | **APC (95% CI)** | **P** | **Years** | | **APC (95% CI)** | **P** | **Years** | | **APC (95% CI)** | **P** |
| **Overall** | 2004-2018 | 0.3 (0.1-0.5) | 0.003 | 2004-2018 | | 0.4 (0.1-0.6) | 0.008 | -- | | -- | -- |
|  |  |  |  |  | |  |  |  | |  |  |
| **Age** |  |  |  |  | |  |  |  | |  |  |
| 0-14 years (Children) | 2004-2018 | -0.1 (-0.6-0.3) | 0.505 | 2004-2018 | | 0 (-0.8-0.7) | 0.947 | -- | | -- | -- |
| 15-39 years (AYA) | 2004-2018 | 0.4 (-0.1-0.8) | 0.103 | 2004-2018 | | 0.9 (0.1-1.6) | 0.035 | -- | | -- | -- |
| 40-64 years (Adults) | 2004-2018 | 0 (-0.3-0.2) | 0.902 | 2004-2018 | | 0.2 (-0.2-0.7) | 0.247 | -- | | -- | -- |
| 65+ years (Older Adults) | 2004-2018 | 0.6 (0.4-0.9) | <.0001 | 2004-2018 | | 0.4 (0.1-0.6) | 0.007 | -- | | -- | -- |
|  |  |  |  |  | |  |  |  | |  |  |
| **Sex** |  |  |  |  | |  |  |  | |  |  |
| Male | 2004-2018 | 0.3 (0.1-0.5) | 0.005 | 2004-2016 | | 0.3 (0-0.7) | 0.064 | -- | | -- | -- |
| Female | 2004-2018 | 0.3 (0.1-0.5) | 0.014 | 2004-2018 | | 0.6 (0.3-0.9) | 0.001 | 2016-2018 | | -3 (-7.3-1.6) | 0.174 |
|  |  |  |  |  | |  |  |  | |  |  |
| **Race** |  |  |  |  | |  |  |  | |  |  |
| White | 2004-2018 | 0.4 (0.2-0.6) | <.0001 | 2004-2018 | | 0.5 (0.2-0.8) | 0.004 | -- | | -- | -- |
| Black | 2004-2018 | 0.7 (0.2-1.3) | 0.011 | 2004-2018 | | 0.3 (-0.5-1) | 0.43 | -- | | -- | -- |
| Other | 2004-2018 | 1.6 (0.7-2.4) | 0.002 | 2004-2018 | | 1 (0.5-1.6) | 0.001 | -- | | -- | -- |

**Supplemental Table 2: Mortality-to-incidence ratios trends histopathologies across age groups from 2004-2018 (SEER) (APC: Annual Percent Change; 95% CI: 95% Confidence Interval)**

|  | **SEER** | | |
| --- | --- | --- | --- |
|  | **Years** | **APC (95% CI)** | **P** |
| **Age** |  |  |  |
| 0-14 years (Children) | 2004-2018 | -0.3 (-1.1-0.4) | 0.341 |
| 15-39 years (AYA) | 2004-2018 | 1.4 (0.4-2.3) | 0.008 |
| 40-64 years (Adults) | 2004-2018 | 1.3 (0.7-1.8) | <.0001 |
| 65+ years (Older Adults) | 2004-2018 | 1.3 (0.7-1.9) | <.0001 |

**Supplemental Table 3: Mortality trends in specific histopathologies across age groups from 2004-2018 (SEER) (APC: Annual Percent Change; 95% CI: 95% Confidence Interval)**

|  | **SEER** | | | | | |
| --- | --- | --- | --- | --- | --- | --- |
|  | **Trend 1** | | | **Trend 2** | | |
|  | **Years** | **APC (95% CI)** | **P** | **Years** | **APC (95% CI)** | **P** |
|  |  |  |  |  |  |  |
| **0-14 years (Children)** |  |  |  |  |  |  |
| Pediatric High-Grade Glioma | 2004-2018 | 0.8 (-0.9-2.4) | 0.336 | -- | -- | -- |
| Embryonal Tumors | 2004-2007 | 7.8 (-6.1-23.7) | 0.253 | 2007-2018 | -3.5 (-5.3--1.7) | 0.002 |
|  |  |  |  |  |  |  |
| **Overall (15 and older)** |  |  |  |  |  |  |
| Glioblastoma | 2004-2018 | 0.4 (0.02-0.77) | 0.04 | -- | -- | -- |
| Other Glioma | 2004-2018 | 0.31 (-0.08-0.71) | 0.11 | -- | -- | -- |
| CNS Lymphoma | 2004-2018 | -0.24 (-0.76-0.29) | 0.347 | -- | -- | -- |
|  |  |  |  |  |  |  |
| **15-39 years (AYA)** |  |  |  |  |  |  |
| Glioblastoma | 2004-2018 | 1.2 (-0.1-2.5) | 0.072 | -- | -- | -- |
| Other Glioma | 2004-2018 | 1.7 (0.6-2.9) | 0.006 | -- | -- | -- |
| CNS Lymphoma | 2004-2018 | -6.3 (-10.5--1.9) | 0.009 | -- | -- | -- |
|  |  |  |  |  |  |  |
| **40-64 years (Adults)** |  |  |  |  |  |  |
| Glioblastoma | 2004-2018 | 0.1 (-0.3-0.6) | 0.577 | -- | -- | -- |
| Other Glioma | 2004-2018 | 0.8 (0.4-1.3) | 0.001 | -- | -- | -- |
| CNS Lymphoma | 2004-2018 | -2.4 (-4.2--0.7) | 0.011 | -- | -- | -- |
|  |  |  |  |  |  |  |
| **65+ years (Older Adults)** |  |  |  |  |  |  |
| Glioblastoma | 2004-2018 | 0.5 (0.1-1) | 0.025 | -- | -- | -- |
| Other Glioma | 2004-2018 | -0.8 (-1.4--0.2) | 0.015 | -- | -- | -- |
| CNS Lymphoma | 2004-2018 | 1.2 (0.7-1.8) | <.0001 | -- | -- | -- |
